# The generation of visual inferences in normal elderly- Influence of schooling and visual complexity

**DOI:** 10.1590/S1980-57642010DN40300194

**Published:** 2010

**Authors:** Ariella Fornachari Ribeiro, Maria Isabel d’Ávila Freitas, Márcia Radanovic, Letícia Lessa Mansur

**Affiliations:** 1 Speech Pathologist, Specialist in Neurolinguistics, Speech Pathology Course, Faculty of Medicine, University of São Paulo (FMUSP), São Paulo SP, Brazil.; 2 Speech Pathologist, Specialist in Neurolinguistics, Speech Pathology Course, FMUSP.; 3 MD, MSc, PhD, Department of Neurology, FMUSP.; 4 MSc, PhD, Assistant Professor. Department of Physiotherapy, Speech Pathology and Occupational Therapy, FMUSP.

**Keywords:** visual inference, normal elderly, visual and inferential complexity

## Abstract

The generation of inferences makes the construction and comprehension of discourse easier, and integrates representations which add coherence to the arguments. Visuoperceptual and inferential deficits merge in the attempt to explain the difficulties that some individuals have in the comprehension of certain kinds of visual stimuli.

**Objectives:**

a) To examine the performance of cognitively healthy elderly subjects in the execution of visual inferences using pictures of different levels of complexity;
b) To compare the performance of subjects according to schooling level.

**Methods:**

A total of 45 normal elderly aged from 61 to 82yrs (M=68; SD=0.57) were examined. The subjects were divided into three groups according to schooling level: Group 1 (1 to 4 years); Group 2 (5 to 8 years) and Group 3 (9 or more years). Each subject had to create a narrative based on four figures with controlled visual complexity. The narratives were transcribed, analysed and scored.

**Results:**

For the essential inferences, the high educated group (3) had a better performance in both visually simple and complex conditions. On the visually complex figures, the medium educated group (2) was statistically equivalent to the high educated group for one figure and equivalent to the less educated group (1) for the other. There was no difference among the groups for the accessory propositions.

**Conclusions:**

Visual complexity interferes with the subject’s ability to make inferences in low and medium educated individuals. High educated subjects maintain the same performance in making inferences, regardless of the visual complexity level.

## Abstract (pt)

A geração de inferências facilita a construção e a compreensão do discurso, e integra representações que dão maior coerência aos argumentos. Os déficits visuoperceptuais e inferenciais confundem-se na tentativa de explicar as dificuldades de alguns indivíduos em compreender alguns tipos de estímulos visuais.

**Objetivos:**

a) Investigar o desempenho de indivíduos idosos normais, na realização de inferências visuais, a partir de figuras de diferentes graus de complexidade visual;
b) Comparar o desempenho dos sujeitos de acordo com os níveis de escolaridade.

**Métodos:**

Foram examinados 45 idosos normais com idades entre 61 e 82 anos (M=68; DP=0.57). Constituiu-se 3 grupos segundo nível de escolaridade: grupo 1 (de 1 a 4 anos); grupo 2 (de 5 a 8 anos) e grupo 3 (9 ou mais anos). A cada indivíduo foram apresentadas 4 figuras, com graus de complexidade visuoespacial controlados para elaboração de discurso. Os itens colhidos foram transcritos, analisados e pontuados.

**Resultados:**

No que diz respeito às proposições essenciais, o grupo de maior escolaridade (3) apresentou melhor desempenho na descrição de ambas as figuras (simples e complexas). Nas figuras visualmente complexas, o grupo com escolaridade média (2) apresentou resultados estatisticamente equivalente ao grupo de alta escolaridade para uma figura e resultado estatisticamente equivalente ao grupo de menor escolaridade (1) para outra figura. Em relação às informações acessórias, não houve diferenças entre os grupos.

**Conclusões:**

A complexidade visual interfere na capacidade de realizar inferências nos indivíduos com baixa e média escolaridade. Indivíduos com alta escolaridade mantêm o mesmo desempenho na realização de inferências, independentemente do grau de complexidade das figuras.

A message conveyed through images stands out from the textual message because it is universal linguistic information of immediate understanding, being understood by people with different languages and cultures. The processing of these images requires the use of linguistic inferences that are directly associated to an individual’s cultural and academic background.^1^

Inferences can be regarded as mental representations made by the subject, through the integration between explicit linguistic information and semantic knowledge.^2,3^ Adequate inferential processing depends on the activation of bilateral large-scale cognitive networks,^4^ including those areas related to visual processing. Visual stimuli are used in most language tests to evoke discourse generation. However, little is known about the criteria adopted in the selection of pictures for this purpose.

Picture description tasks evaluate how fast and accurately the subject can interpret extralinguistic and contextual information.^5^ In addition, the generation of inferences facilitates the construction and comprehension of discourse, fills the gaps of available information and integrates representations that give continuity and coherence to the arguments.^2,6^

For this whole circuit to complete properly, the cortex is activated with information coming from the long term semantic memory, which will promote an organized discursive structure during the inferential process.^4^

Therefore, from the beginning of the inferential processing, there is in an organized order, the semantic encoding and the creation of mental models, culminating in the proper understanding of the stimulus ideas.^7,8^ These mental models involve a set of propositions that combine implicit and explicit stimulus information, which are culturally determined and learned from our experience in society.^9^

Furthermore, studies report that areas from both hemispheres are involved in this process, each area accounting for different conditions and levels of inferential processing.^10,11^ These conditions involve the complexity of visual stimuli presented, the individual’s familiarity with the stimulus, the stimulus frequency (high or low), and its contextualization, among others. This means that depending on the presented stimulus and its characteristics, the inferential processing will occur in different levels and brain regions, and the verbal expression of these individuals will also occur differently.^12^

Studies such as that of Kintsch and Van Dijk^13^ that included a production model through analysis and synthesis, called a situational model, described that the thematic propositions that are essential to the comprehension of a stimulus are stored in working memory longer than those considered less important. Kintsch and Van Dijk termed relevant propositions “macropropositions”, while the thematic structure is called “macrostructure”.

This model reinforces the idea that the various areas addressing the issue of stimuli comprehension and discourse production indicate a trend of research in the importance of prior knowledge, and the strategies for transforming information into knowledge, and contextual variables, among others.^13^

It is well known that schooling also interferes directly in skills involving perceptual and inferential tasks.^12,14^ Among the losses commonly associated with aging is the decline in cognitive function, particularly regarding memory and speed of information processing.^15-17^ However, the effect of education may lead to a false impression of cognitive decline in people with little education.^18,19^

In this perspective, the visual comprehension of stimuli is important for proper verbal expression, and the visual complexity of the material presented interferes with the subject’s performance.^12^ Visuoperceptual and inferential deficits merge in the attempts to explain the impairment that some individuals have in comprehending certain kinds of visual stimuli. However, we do not know which of these deficits plays the major role in discourse production.

The interest in the neuropsychological processes among the normal population is increasing and this issue is becoming more and more frequently the focus of research in Psychology and Linguistics, in order to detect possible language modifications that might be due to the aging process in its interaction with cultural aspects.^20,21^ In a previous study, our group verified that the elderly subjects in our sample had an intriguing difficulty in naming actions when the stimuli were presented as pictures. In this task, age had a negative impact on the subjects’ performance.^22^

Based on the data outlined, this study aimed to:

a) Investigate the performance of normal elderly on tasks of visual inference using pictures of different degrees of visual complexity;
b) Compare the performance of subjects according to schooling level.

## Methods

### Casuistics

A total of 45 normal elderly subjects (24 females and 21 males) were examined. The sample was divided into three groups according to educational level: Group 1 (1 to 4 years); Group 2 (5 to 8 years) and Group 3 (9 and above).

### Inclusion criteria

All subjects enrolled in this study signed a written inform consent (CAPpesq o nº 701/06). All participants had to be aged 60 or older and have Brazilian Portuguese as their mother tongue. Subjects were included only if they had normal scores (for the Brazilian population) on the following batteries:

- Mini-Mental State Examination (MMSE):^23^ the cutoff score was 23/24 for subjects with 4 to 8 years of formal education; 25/26 for subjects with 8 to 11 years of education; 27/28 for subjects with more than 11 years of education.
- Pfeffer Functional Activities Questionnaire (PFAQ):^24^ the cutoff score was 4/5.
- Informant Questionnaire on Cognitive Decline in the Elderly (IQCODE):^24^ the cutoff score was 3.41.

### Exclusion criteria

Subjects were excluded if one or more of the following conditions were present: previous language disorder, past / present history or clinical evidence of neurological disease that could impair perceptual or cognitive functions (such as cerebrovascular disease, Parkinson’s disease), past / present history or clinical evidence of psychiatric disease, use of alcohol or drugs that could interfere with mental function, and visual or auditory impairment that could interfere with the subject’s performance on the tests.

### Instrument

The pictures were taken from a pool of illustrations chosen by a consensus of 15 speech therapists to compose a set of four pictures drawn in black and white, targeting an adult audience, and representing scenes that differed from each other in visual complexity (number of objects in each picture) but were similar in inferential complexity (level of inferential processing necessary to understand the scene). Caricatures and stylized drawings were excluded. The pictures were divided into two groups, each composed by two scenes:

1. Visually simple/Inferentially simple (VSIS);
2. Visually complex/Inferentially simple (VCIS).

**Figure 1:**
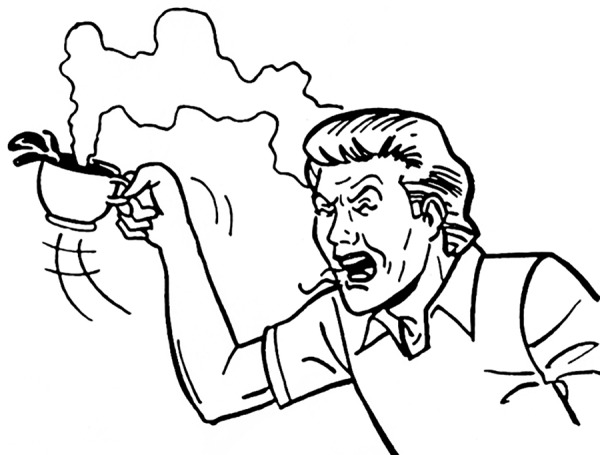
VSIS (Visually simple and inferentially simple).

**Figure 2:**
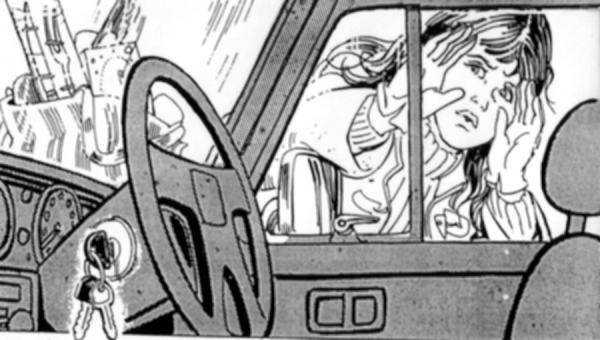
VCIS (Visually complex and inferentially simple).

All pictures represented actions (drinking, smoking, running, looking). The complexity was determined by the number of items, according to the criteria defined by Myers.^12^ A visually simple picture had fewer items and required less interpretation (Figure 1), the opposite being true for a visually complex picture (Figure 2). Each figure presented four essential propositions that were supposed to be made by the subjects. Each proposition scored two points, giving a total of 8 points per figure.

### Procedure

Subjects were tested individually, and were told to look carefully at the pictures and describe what was happening in each of them. There was no time restriction to perform the task. All narratives were tape-recorded and transcribed by the first author for later analysis.

### Data analysis

The same group of 15 judges mentioned in the Instrument Section acted as judges in order to establish the number of items of essential information for each picture and to agree upon the minimum score for each one. The items obtained from the subjects’ transcriptions were analyzed and scored according to an adapted version of Joanette’s proposal for the evaluation of discursive abilities based on pictures.^25^ This study used a model of analysis called “propositional analysis” in which the discourse is analyzed according to the number of propositions, i.e. information that is emitted during the generation of an oral narrative from a pictorial history. The author divides propositions into “simple” and “complex”, corresponding to essential and accessory propositions in our study. Essential propositions correspond to the inferential production itself. Thus, to discriminate these propositions from other types of information, the following scores were created:

1. Essential information: minimal information that allow the development of the description; these are essential to comprehension (2 points each);
2. Accessory information: pertinent information, which add relevant data, but are not necessary for the comprehension; they add accuracy or explain data that can be inferred (1 point each);
3. Non-pertinent information: personal interpretation; items that do not belong to the original scene, but that are not incoherent nor change the meaning of the scene (0.5 point each);
4. Incoherent information: items that, whether related to the scene or not, reveal lack of understanding, difficulty in reporting the elements, or ruptures; these can be intrusions, elements from other stories or confabulations (0.5 point each).

Furthermore, the total number of words emitted by the subjects during the discourse production was computed. Examples of the figures and the narrative production of the subjects and their scores are displayed in the Appendix.

### Statistical analysis

The number of essential, accessory, non-pertinent, incoherent, and total propositions was computed for each group. The scores obtained for each type of proposition in all figures, the performance on each level of inferential processing, and the number of words used in each description (verbosity index) were compared searching for intergroup differences. The Kruskal-Wallis Test with Dunn’s post test was performed for all comparisons. The level of significance adopted was 5%. The statistical software used was MedCalc® version 11.0.1.

## Results

As shown in Table 1, there were no significant differences in the subjects’ demographic variables regarding gender and age, except for educational level, as expected. Tables 2, 3 and 4 show that the groups performed differently in the generation of inferences, according to schooling, both on VSIS and VCIS pictures. Lower educated subjects performed worse on making essential inferences and in terms of total scores than did higher educated subjects for both visually simple and complex pictures. There were no differences among the groups in making accessory, non-pertinent and incoherent inferences. In two groups (1 and 3), there was a statistically significant difference in the number of words generated (verbosity index) by the highest educated group compared to the lowest educated group for the narrative production, but this difference was not found in Figures 2 (Table 2 and 3). Intragroup comparison showed that visual complexity exerted a powerful negative effect on the lowest educated group, which performed poorer on the VCIS pictures (Table 4).

**Table 1:** Demographic characteristics of the sample.

Group	G1			G2			G3		p (two-tailed)
	**M (SD)**	**Range**		**M (SD)**	**Range**		**M (SD)**	**Range**	
Age (yrs)	68.4 (4.2)	61-75		69.8 (5.8)	61-82		68.4 (7.3)	61-85	0.758
Schooling (yrs)	3.2 (1.3)	1-4		7 (1.2)	5-8		12.8 (2)	11-15	< 0.0001
Gender M F	7 8			6 9			7 8		0.913

M: mean; SD: standard deviation.

**Table 2:** Comparison among groups in making of inferences for Figures 1 and 2 (Visually simple and inferentially simple - VSIS).

Figures	G1			G2			G3		p (two-tailed)	Multiple comparison
	**M (SD)**	**Range**		**M (SD)**	**Range**		**M (SD)**	**Range**		
Figure 1										
Essential inf.	3 (1.6)	0-6		3.5 (1.2)	2-6		5 (1.8)	2-8	0.007	1-3 (< 0.01)
Accessory inf.	0.5 (0.6)	0-2		0.8 (0.3)	0-1		1.3 (1.3)	0-5	0.097	NA
Total inf.	3.6 (1.9)	0-8		4.3 (1.3)	2-7		6.3 (2.5)	3-11	0.006	1-3 (< 0.01)
Non-pertinent inf.	0.1 (0.2)	0-0.5		0 (0)	0-0		0 (0)	0-0	0.129	NA
Incoherent inf.	0.1 (0.3)	0-4		0.3 (0.4)	0-1.5		0.1 (0.2)	0-0.5	0.229	NA
Verbosity index	14.1 (9.6)	5-44		17.7 (8)	10-44		24.8 (15)	6-61	0.013	1-3 (< 0.05)
Figure 2										
Essential inf.	3.3 (2.5)	0-8		3.6 (2.4)	0-8		5.7 (1.7)	2-8	0.009	1,2-3 (< 0.05)
Accessory in	1.1 (0.9)	0-2		0.6 (0.5)	0-1		0.9 (1.1)	0-3	0.326	NA
Total inf.	4.5 (2.7)	0-8		4.2 (2.5)	0-9		6.7 (2.3)	3-11	0.023	2-3 (0.009)
Non-pertinent inf.	0.2 (0.3)	0-1		0.4 (0.7)	0-2.5		0.1 (0.2)	0-0.5	0.196	NA
Incoherent inf.	0.2 (0.5)	0-1.5		0.3 (0.4)	0-1		0.1 (0.4)	0-1.5	0.150	NA
Verbosity index	19.6 (6.7)	6-33		24 (9.6)	10-44		28.4 (13)	16-60	0.236	NA

M: mean; SD: standard deviation; NA: not applicable.

**Table 3:** Comparison among groups in making of inferences for Figures 1 and 2 (Visually complex and inferentially simple - VCIS).

Figures	G1			G2			G3		p (two-tailed)	Multiple comparison
	**M (SD)**	**Range**		**M (SD)**	**Range**		**M (SD)**	**Range**		
Figure 3										
Essential inf.	0.7 (1.2)	0-4		2.7 (1.6)	0-6		5.2 (2.6)	0-8	< 0.0001	1-2 (< 0.05); 1-3 (< 0.001)
Accessory inf.	1 (0.9)	0-3		1.3 (0.7)	0-3		1 (1.5)	0-4	0.387	NA
Total inf.	1.7 (1.5)	0-5		3.9 (1.7)	1-7		6.3 (2.8)	0-12	< 0.0001	1-2 (< 0.05); 1-3 (< 0.001)
Non-pertinent inf.	0.2 (0.8)	0-3		0 (0.1)	0-0.5		0 (0.1)	0-0.5	0.998	NA
Incoherent inf.	0.4 (0.4)	0-1		0.5 (0.6)	0-2		0.2 (0.4)	0-1.5	0.152	NA
Verbosity index	15.4 (6.7)	3-30		22 (9.4)	9-36		33.2 (23.1)	7-88	0.023	1-3 (< 0.05)
Figure 4										
Essential inf.	0.9 (1.5)	0-4		2.7 (1.8)	0-8		5.9 (2.7)	0-8	< 0.0001	1-3 (< 0.001); 2-3 (< 0.05)
Accessory in	0.5 (0.6)	0-2		0.8 (1.2)	0-4		0.4 (0.7)	0-2	0.548	NA
Total inf.	1.5 (1.9)	0-6		3.3 (2.1)	0-8		6.3 (2.6)	0-8	< 0.0001	1-3 (< 0.001); 2-3 (< 0.05)
Non-pertinent inf.	0 (0)	0		0.1 (0.2)	0-0.5		0 (0.1)	0-0.5	0.351	NA
Incoherent inf.	0.6 (0.7)	0-2		0.3 (0.6)	0-2		0.2 (0.4)	0-1.5	0.196	NA
Verbosity index	17.8 (9.6)	3-41		19.8 (6.8)	11-35		26.1 (10.7)	13-46	0.072	NA

M: mean; SD: standard deviation; NA: not applicable.

**Table 4:** Comparison among groups in the making of inferences for visually simple and inferentially simple (VSIS) and visually complex and inferentially simple (VCIS) figures.

Group	VSIS			VCIS		p
	**M (SD)**	**Range**		**M (SD)**	**Range**	
1	4 (2.3)	0-8		1.6 (1.7)	0-6	< 0.0001
2	4.2 (1.9)	0-8		3.6 (1.9)	0-8	0.141
3	6.5 (2.4)	3-11		6.2 (2.6)	0-12	0.626

## Discussion

The main purpose of our study was to verify the effect of schooling on the process of inference generation in a sample of elderly people, and this was achieved by controlling the visual and inferential complexity of the pictures used as stimuli.

In the inference processing involving visually and inferentially simple pictures, we observed that only the highly educated group (Group 3) had better performance in describing the pictures. However, in the visually complex pictures, the three groups performed differently. With regard to the essential propositions, the higher educated group (3) performed better in the description of both figures (simple and complex). For the visually complex figures, the medium educated group (2) performed similarly to the group with high schooling level on one figure and similarly to the group with lower schooling level (1) on the other figure. In relation to accessory information, there were no differences among the groups. With regard to the non-pertinent and incoherent propositions, the three groups behaved similarly.

Previous studies report that, in elderly subjects with an average schooling of 14 years, visual complexity does not interfere with picture description or inference generation abilities.^12^ These subjects had a slightly lower performance in the generation of inferences for visually complex pictures compared to visually simple pictures, but this difference was not statistically significant. Our findings are in accordance with the data available not only for the highly educated individuals (over 11 years of schooling), but also for the lower and intermediate educated groups (Groups 1 and 2). In the latter, we noted lower performance of the less educated group (Group 1), even on those pictures which were visually simple. This group comprises subjects who, in most cases have basic reading skills, but do not use these on a daily basis or for professional purposes. Our results confirmed previous findings regarding the effect of schooling on verbal tasks that demand cognitive processing.^26-28^

The fact that we did not find any differences between the groups when considering the accessory propositions indicates that it is not necessary to subdivide the discourse when scoring the task. We did not find any studies dealing with this issue. Therefore, we hypothesize that this finding is due to the fact that these propositions do not require complex integrations and associations, as do those involved in the inferential process itself. This information is merely a complement, and occurs independently to adequate inferential processing. Thus, not only does educational level play no part in it, but also the number of accessory propositions does not improve the subjects’ ability to make inferences.

A study involving oral discourse production from graphic stimuli showed that the essential propositions remained in memory longer than accessory information. In the cited study, even after a time interval, the subjects failed to emit accessory propositions during the discourse production. The subjects did however, maintain the emission of macropropositions and the general text macrostructure.^13^ Our findings corroborate these data.

An important point to be highlighted is that our results suggest that the two visually simple (1 and 2) and complex (3 and 4) figures do not seem to be equivalent. This becomes evident especially on the analysis of the performance of the medium educated group, which resembled either the higher or the lower educated groups in figures with the same degree of complexity. These results can be partially explained by the process of picture selection. The lack of standardized pictorial material and inferential tests in Brazilian Portuguese required the selection of figures according to an expert consensus, but these figures were not the subject of any prior pilot study.

Regarding the total number of words emitted by the subjects, our results showed that as educational level improved, the number of words emitted also increased and there was a *continuum* among the groups. However, significant differences were found only between Groups 1 and 3 on one visually simple and one visually complex figure. These data also point to the fact that the two figures in the same category might not be perfectly equivalent, as previously mentioned. Moreover, the computation of non-relevant and incoherent propositions, in which the three groups performed similarly, suggested that the subjects’ narratives were equivalent in pertinent content and, therefore schooling seemed to exert a positive effect on the verbosity index.

A narrative or coherent report is a complex cognitive task that requires good organization and planning of language aspects including word and sentence ordering, correct use of articles, pronouns and conjunctions, as well as concordance and conjugation of verbs. These elements constitute the oral discourse cohesion, enabling the narrative to have relevance according to the proposed theme.^21^ The elderly may acquire a high degree of cognitive expertise, derived from cultural influence, which allows them to overcome the biological limitations of aging as well as to compensate for educational deficits and environmental hazards.^21^

There is a well known relationship between the ability to comprehend and produce the macrostructure of the discourse and the ability to make inferences, with various levels of processing ranging from perception of the individual items to integration and association of relevant clues to previous experience.^5^

Most studies disclose the central importance of the context, understood as a collection of suppositions (information) that comes to the subjects’ mind as the inferential process occurs. These suppositions may come from the environment (through the linguistic code or perceptual stimuli), from the long-term semantic memory (which stores our knowledge and beliefs) and from deductive reasoning. These suppositions are then interpreted, leading to valid conclusions, by means of intuitive, qualitative and comparative judgments.^29^ In other words, the context will contribute allowing the subject to adequately go through each step of the inferential process, allowing correct inferences to be made.

The influence of schooling in the generation of inferences is conditioned by different types of context, as follows:

- Cultural context, formed by cultural principles and the influence of knowledge within the limits of the particular representational units and the extracted inferences;^30^
- Situational context, the circumstance of action, by which the subject’s intentions and perspectives are affected, and that contributes to stimulus processing;
- And principally, the personal context, which includes the knowledge, attitudes, and emotional characteristics of the individual. For instance, subjects apprehend the meaning of a text by analyzing the details of the stimuli, linguistic rules and conventions in general, which in turn are conditioned by several elements: gender, age, education, occupation, etc.

Our findings can be summarized as follows:

- Visual complexity interfered with the low and medium educated subjects’ ability to make inferences: the more complex the picture, the lower the performance in making inferences.
- Highly educated subjects maintained the same effectiveness in the making inferences, independently of the picture complexity. The process of inference generation in these subjects was superior for all pictures compared to low and medium educated subjects.
- Our results suggest a *continuum* in inferential processing, which is directly related to the schooling level.
- In relation to the accessory information, there was no influence of schooling level on performance of the subjects.

These data should be taken into account in the devising and/or selection of visual instruments for evaluation purposes in neuropsychological testing using visual material, or in the selection of visual material as a source of information for the public in general (such as signals and signboards), since these variations in interpretation related to schooling level probably occur in daily life situations.

## APPENDIX

### Examples of narratives based on the inferential processing and the respective scores. In all examples, subjects had the same schooling level (Group 3-high).

#### Example 1

“Tem um homem com um vaso de plantas ou um café. É um vaso que estava no suporte”

[There is a man with a flowerpot or a coffee. It is a pot that was on a support.]

Scoring:

- Essential propositions: 0
- Accessory propositions: 0
- Non pertinent propositions: 0
- Incoherent propositions: 1
- Total number of words: 18 (in Portuguese)

#### Example 2

“Aí tem um homem. Ele está sozinho. Ele está vestindo uma camisa pólo aberta no pescoço. Tem uma xícara em sua mão, que tem um líquido escuro que parece ser café, e está derramando porque ele sacode a xícara. Poderia se deduzir que ele colocou a xícara nos lábios para beber. Pela sua expressão de dor, ele deve ter queimado a boca.”

[There is a man. He is alone. He is wearing a polo shirt open at the neck. He has a cup in his hand, which has a dark liquid that appears to be coffee, and it is spilling out because he is shaking the cup. One could deduce that he placed the cup against his lips to drink. By his expression of pain, he must have burnt his mouth.]

Scoring:

Essential propositions: 8
Accessory propositions: 3
Non pertinent propositions: 0
Incoherent propositions: 0
Total number of words: 61 (in Portuguese)

#### Example 1

“Aqui está uma chave pra abrir o porta-malas. A menina vai tirar a chave pra abrir atrás pra pegar alguma coisa.”

[Here is a key to open the trunk. The girl will get the key to open up the back and get something out.]

Scoring:

- Essential propositions: 0
- Accessory propositions: 1
- Non pertinent propositions: 0
- Incoherent propositions: 1
- Total number of words: 22 (in Portuguese)

#### Example 2

“É o interior de um carro. É um carro inglês porque o volante está do lado direito. A chave está na ignição. A pessoa que saiu bateu a porta com a chave lá dentro. O rosto é de uma menina, ela ta do lado de fora, com as mãos no vidro, olhando pra dentro. Tem compras no capô do carro, uma cartela com garfo e faca. Ela deve estar se perguntando “como será que eu vou entrar se a porta está trancada e a chave está lá dentro?”

[It is the interior of a car. It is an English car because the steering wheel is on the right. The key is in ignition. The person who left slammed the door with the key inside. The face is of a girl, she is outside, her hands on the glass, looking inside. There are shopping items on the hood of the car, a card with a knife and a fork. She must be asking herself “how will I get in if the door is locked and the key is in there?]

Scoring:

Essential propositions: 8
Accessory propositions: 4
Non pertinent propositions: 0
Incoherent propositions: 0
Total number of words: 61 (in Portuguese)
